# Raltitrexed–eloxatin salvage chemotherapy in gemcitabine-resistant metastatic pancreatic cancer

**DOI:** 10.1038/sj.bjc.6603026

**Published:** 2006-02-28

**Authors:** M Reni, L Pasetto, G Aprile, S Cordio, E Bonetto, S Dell'oro, P Passoni, L Piemonti, C Fugazza, G Luppi, C Milandri, R Nicoletti, A Zerbi, G Balzano, V Di Carlo, A A Brandes

**Affiliations:** 1 Department of Oncology, S. Raffaele Hsc. Scientific Inst., via Olgettina 60, 20132 Milan, Italy; 2 Department of Oncology, Azienda Ospedaliera and University, Padova, Italy; 3 Department of Oncology, Policlinico Universitario, Udine, Italy; 4 Department of Oncology, Garibaldi-Nesima H. Catania, Catania, Italy; 5 Department of Radiology, S. Raffaele Hsc. Scientific Inst., Milan, Italy; 6 Laboratory of Experimental Surgery, Department of Diabetes and Transplant Immunology, S. Raffaele Hsc. Scientific Inst., Milan, Italy; 7 Department of Medical Oncology, Azienda Ospedaliera-Policlinico, Modena, Italy; 8 Department of Oncology, Pierantoni H. Forlì, Forlì, Italy; 9 Department of Surgery, S. Raffaele Hsc. Scientific Inst., Milan, Italy

**Keywords:** chemotherapy, metastatic disease, oxaliplatin, pancreatic cancer, raltitrexed, salvage therapy

## Abstract

Limited information on salvage treatment in patients affected by pancreatic cancer is available. At failure, about half of the patients present good performance status (PS) and are candidate for further treatment. Patients >18 years, PS ⩾50, with metastatic pancreatic adenocarcinoma previously treated with gemcitabine-containing chemotherapy, and progression-free survival (PFS) <12 months received a combination of raltitrexed (3 mg m^−2^) and oxaliplatin (130 mg m^−2^) every 3 weeks until progression, toxicity, or a maximum of six cycles. A total of 41 patients received 137 cycles of chemotherapy. Dose intensity for both drugs was 92% of the intended dose. Main grade >2 toxicity was: neutropenia in five patients (12%), thrombocytopenia, liver and vomiting in three (7%), fatigue in two (5%). In total, 10 patients (24%) yielded a partial response, 11 a stable disease. Progression-free survival at 6 months was 14.6%. Median survival was 5.2 months. Survival was significantly longer in patients with previous PFS >6 months and in patients without pancreatic localisation. A clinically relevant improvement of quality of life was observed in numerous domains. Raltitrexed–oxaliplatin regimen may constitute a treatment opportunity in gemcitabine-resistant metastatic pancreatic cancer. Previous PFS interval may allow the identification of patients who are more likely to benefit from salvage treatment.

Pancreatic adenocarcinoma has a dismal prognosis due to early metastatic dissemination even in patients submitted to surgery with radical intent. As a consequence, effective systemic treatment has a strategic role in the therapeutic management of this disease. Unfortunately, very few agents have demonstrated any activity with reproducible response rates greater than 15%. While randomised studies have suggested that chemotherapy is superior to best supportive care in prolonging survival and improving symptoms in patients with advanced disease (Glimelius *et al*, 1996), standard single agent gemcitabine yields a marginal impact on disease outcome. Median progression-free survival (PFS) with this agent is approximately 3 months (Burris *et al*, 1997; Bramhall *et al*, 2001, 2002; Berlin *et al*, 2002; Moore *et al*, 2003; Rocha Lima *et al*, 2004; Van Cutsem *et al*, 2004; Reni *et al*, 2005), and <15% of patients are PF at 6 months (PFS-6) from diagnosis (Reni *et al*, 2005). Approximately half of the patients failing previous treatment present good performance status (PS) and are willing to undergo further treatment. However, very limited information concerning the impact of salvage treatment upon survival and quality of life is available. This patient population represents the target for experimental trials aimed at broadening the chemotherapeutic armamentarium. Raltitrexed (Tomudex® AstraZeneca S.p.A., Ben Venue Laboratories Inc., Bedford, OH, USA) is a thymidylate synthase inhibitor that is easily transported in the cell, where it undergoes extensive polyglutamation within the cells, which extends the intracellular retention, increases concentration, and ultimately leads to increased cytotoxicity. Raltitrexed blocks the production of thymidine monophosphate from deoxyuridine monophosphate in a reaction-specific manner. Oxaliplatin (Eloxatin®, Sanofi-Synthelabo S.p.A, Milan, Italy), a third-generation platinum analogue, is a diaminocyclohexane platinum that forms interstrand DNA adducts, which differ from those formed by cisplatin or carboplatin in their capability to overcome resistance mechanisms. Preclinical studies suggested that pancreatic cancer cell lines are highly sensitive towards raltitrexed and oxaliplatin even in gemcitabine- and 5-fluorouracil-resistant cells (Kornmann *et al*, 2000; Monti *et al*, 2004), and that oxaliplatin yields an addictive antitumour activity when combined with raltitrexed or other thymidylate synthase inhibitors (Raymond *et al*, 1998), thus encouraging the use of these two drugs in experimental protocols as salvage treatment (Monti *et al*, 2004). Furthermore, raltitrexed and oxaliplatin have a noncrossresistant mode of action, differential toxicity profiles, and can be used in combination as outpatient therapy in the same doses as for single agent use (Fizazi *et al*, 2000).

Raltitrexed-oxaliplatin (TOM-OX) combination has been assessed in colorectal cancer and mesothelioma yielding elevated tumour control rates in 5-fluorouracil or cisplatin pretreated patients (Cascinu *et al*, 2002; Seitz *et al*, 2002). Single agent raltitrexed obtained 5% partial response (PR) and 29% stable disease (SD) in 42 patients with pancreatic adenocarcinoma (Pazdur *et al*, 1996).

A multicentre phase II trial was undertaken to determine the activity and safety of TOM-OX combination as salvage treatment in gemcitabine-resistant metastatic pancreatic cancer.

## PATIENTS AND METHODS

### Patient population

Patients aged >18 years with histologically or cytologically proven metastatic pancreatic adenocarcinoma, with at least one bidimensionally measurable target lesion were eligible for this study. Patients were to have received previous gemcitabine-containing chemotherapy. No definition of gemcitabine resistance in pancreatic cancer exists and no other trial has used this eligibility criterion. Thus, in the absence of benchmarks to which to refer, it was arbitrarily decided to include only those patients in whom progression occurred <12 months from the start of treatment (i.e. <6 months from treatment conclusion) as it was deemed unlikely that these patients could achieve relevant benefit with further gemcitabine administration. Other inclusion criteria were: Karnofsky PS⩾50, adequate bone marrow (absolute neutrophil count (ANC) ⩾1500 cells mm^−3^, platelet count ⩾100 000 cells mm^−3^, and haemoglobin ⩾10 g dl^−1^); kidney function (creatinine clearance ⩾65 ml min^−1^) and liver function (serum total bilirubin ⩽2 mg dl^−1^, alkaline phosphatase and serum transaminases ⩽three times the upper limit of normal (ULN)). Patients with prior malignancy were ineligible for the study, with the exception of those who had had basal-cell carcinoma of the skin, carcinoma *in situ* of the cervix, or other cancer for which the patient had been disease free for at least 5 years. Patients with ampullary tumours or other histologic variants of pancreatic carcinoma were ineligible. The study was reviewed and approved by each local Ethics Committee of the participating institutions and was conducted in accordance with the Declaration of Helsinki. All participating patients were required to provide written informed consent.

### Treatment plan

Raltitrexed was diluted in 5% dextrose and given as 15 min intravenous (i.v.) infusion at 3 mg m^−2^. After a 45 min interval, oxaliplatin was administered in at least 2 h i.v. infusion at 130 mg m^−2^. Patients were systematically given prophylactic antiemetic treatment with 5-HT3 antagonists. Cycles were repeated every 3 weeks until PD, unacceptable toxicity, patient's or physician's decision, or a maximum of six cycles. Dose adjustments were made according to the greatest degree of toxicity. In the case of ANC <1500 cells mm^−3^, of platelet count <100 000 cells mm^−3^, or of ⩾grade 3 nonhaematological toxicity, on the first day of the next cycle, the treatment was withheld until recovery and then restarted with dose for the drug responsible for nonhaematological toxicity reduced by 25%. If recovery was not evident within 2 weeks, the patient was discontinued from the study. If grade 3 or grade 4 haematological toxicity occurred, doses for both drugs were reduced by 25% or by 50%, respectively. Treatment was discontinued in cases of grade 4 haematological toxicity associated with grade 3 gastro-intestinal toxicity. If grade 2 or grade 3 gastro-intestinal toxicity occurred, raltitrexed dose was to be reduced by 25% or by 50%, respectively. Treatment was discontinued in cases of grade 4 gastro-intestinal toxicity. In cases of decreased creatinine clearance, raltitrexed was administered every 4 weeks at 75% (55–65 ml min^−1^) or 50% (25–54 ml min^−1^) of the original dose. Raltitrexed was discontinued if creatinine clearance fell below 25 ml min^−1^. The oxaliplatin dose was to be reduced to 100 mg m^−2^ in cases of paraesthesia or dysesthesia with pain or functional impairment lasting >7 days, to 80 mg m^−2^ for persistent paraesthesia or dysesthesia between two cycles without functional impairment, or discontinued in cases with persistent paraesthesia or dysesthesia between two cycles with functional impairment.

### Study evaluations

Pretreatment evaluation consisted of PS assessment, haematological and biochemical profiles, CA 19-9 analysis, spiral computed tomography (CT) scan of the abdomen, and the chest or magnetic resonance imaging (MRI). During treatment, blood chemistry, creatinine clearance, and CA 19-9 analysis was performed on day 14, whereas haematological profile was repeated on day 1 of every cycle. Imaging studies, employing the same method used to measure the initial target, were repeated every two treatment cycles to assess objective response. At the end of chemotherapy, CA 19-9 analysis was performed every 40–50 days, and imaging studies were repeated every 2–3 months, when an increase of CA 19-9 was observed, or when PD was suspected. The EORTC QLQ-C30 (Aaronson *et al*, 1993) and PAN26 (Fitzsimmons *et al*, 1999) questionnaires for quality of life (QOL) assessment were given to patients at study entry and every second cycle of chemotherapy, until PD.

### Outcome measures

Side effects were graded according to the Common Toxicity Criteria defined by the NCI (US), extended by the NCIC (Canada) version 2.0 (Ajani *et al*, 1990). The objective tumour response to treatment was assessed according to the WHO criteria on the basis of a maximum of three ‘target lesions’ selected before the start of the treatment. All scans were centrally reviewed by one expert radiologist. The duration of complete response was defined as the time between the first documentation of complete disease resolution and the first documented observation of PD. The duration of PR was defined as the time between the initiation of treatment and the time of PD. The PFS was defined as the interval between the initiation of treatment and the occurrence of PD. Survival (OS) was measured from the initiation of treatment to the date of death for any reason or to the last follow-up assessment. The QOL was assessed using the EORTC QLQ-C30 questionnaire (Aaronson *et al*, 1993) supplemented by the pancreatic cancer module (EORTC QLQ-PAN-26) (Fitzsimmons *et al*, 1999). Differences >10 points on the transformed scales were regarded as clinically significant (Osoba *et al*, 1998). Mean scale and items scores were transformed to a 0–100 scale, as described in the EORTC scoring manual (Fayers *et al*, 2001). To be assessable for QOL, patients had to have a baseline QOL assessment and at least one subsequent QOL assessment. The numbers of patients in each analysis may differ from scale to scale as some patients may have had randomly missing scores on certain scales.

### Statistical analysis

The primary end point of this trial was to assess the objective response rate of TOM-OX in gemcitabine-resistant metastatic pancreatic adenocarcinoma. Secondary end points were PFS, OS, toxicity, response duration, and QOL. The Simon Minimax two-stage design was used. The maximum response rate considered of low interest was 10% and the minimum response rate considered of interest was 25%. The sample size was calculated with a type I error of 10% and a test power of 90%. Early discontinuation of the study was planned in the case of <3 responses in the first 27 patients. Alternatively, the target enrollment was estimated to be 40 patients. TOM-OX would be considered an active regimen in this patient population if >6 responses were noted among the 40 enrolled patients. All the statistical analyses were performed on the intention-to-treat population. The survivor functions curves were estimated according to the Kaplan–Meier method and compared using the log-rank test. All the probability values were from two-sided tests. Analyses were carried out using the Statistica 4.0 statistical package for Windows (1993 Statsoft, Tulsa, OK, USA).

### Funding source

Raltitrexed and oxaliplatin were supplied gratuitously by Astra-Zeneca, Italy and Sanofi-Synthelabo, Italy. No funding sources supported the work.

## RESULTS

### Patient population

Between December 2002 and March 2004, 41 patients were entered into this trial. The characteristics of the patient population are listed in Table 1. Previous PFS, which was calculated as the interval between the initiation of latest chemotherapy treatment and the occurrence of PD, was 1–11.5 months (median 6). With regard to previous treatment, 16 of 18 patients submitted to surgery with curative intent received postoperative chemotherapy, which was followed by radiotherapy in 10 cases, while two patients were submitted to postoperative chemoradiation and received gemcitabine at the time of first recurrence. Two of the 23 patients who did not receive prior surgery were irradiated. Among 35 patients receiving a single prior chemotherapy, treatment consisted of gemcitabine alone in 17 patients, PEFG (cisplatin, epirubicin, 5-fluorouracil, gemcitabine) regimen (Reni *et al*, 2005) in 16 patients, gemcitabine plus cisplatin or 5-fluorouracil in one case each. Among six patients receiving either two (*n*=5) or three (*n*=1) prior chemotherapy lines, first-line treatment consisted of gemcitabine in all cases and was followed as second-line treatment by PEFG regimen (*n*=5) or 5-fluorouracil and folinic acid (*n*=1); one patient also received mitomycin-C after gemcitabine and PEFG regimen, as third-line treatment. In total, 26 patients had PD during previous chemotherapy, and 15 had an interval <5 months between the end of previous therapy and PD.

### Treatment summary and toxicity

A total of 137 cycles were delivered. Total number of cycles per patient is reported in Table 2. In all, 13 (32%) patients received six cycles, while 28 discontinued treatment due to radiologically confirmed PD (16), clinical PD without radiological assessment (five), patient or medical decision (five), persistent thrombocytopenia (one), and death of heart failure (one). Dose intensity was 92% for both drugs. The mean interval between cycles was 22.8 days. The start of a new cycle was delayed by 7–14 days in 18 cycles (13%) due to persistent neutropenia (*n*=6) or thrombocytopenia (*n*=1), fever (*n*=1), liver toxicity (*n*=2), bowel subocclusive status (*n*=1), delay in CT scan reassessment (*n*=2), patient or medical decision (*n*=5). Raltitrexed dose was reduced in five (12%) patients either by 50% due to G3 vomiting (*n*=1) or by 25% due to grade 2 liver toxicity (*n*=1) or fatigue (*n*=3). Oxaliplatin dose was reduced in four (10%) patients by 25% due to G3 liver toxicity (*n*=1), G2 liver toxicity, or fatigue (*n*=1 each).

Table 3 summarises the main side effects observed. One patient died on day 1 of the third cycle due to heart failure. Febrile neutropenia, or non-neutropenic infections were not observed.

### Response and survival

Table 4 summarises the outcome measures. The central radiology independent review showed 10 PR (24%; 95% confidence interval (95% CI) 11–37%), 11 SD (27%; 95% CI 13–41%), 15 PD (37%; 95% CI 22–52%), while five patients (12%; 95% CI 2–22%) discontinued chemotherapy before tumour assessment due to clinical PD. Median duration of PR was 5.6 months (interquartile range: 4.3–6.4 months) and five of 10 patients with PR were PF at 6 months. Median duration of SD was 4.0 months (interquartile range 3.1–4.5 months) and one of 11 patients with SD was PF at 6 months. Of 35 patients, 13 (37%; 95% CI 20–54%) with elevated CA19.9 basal value had a marker reduction of >50% during treatment.

**Figure 1:**
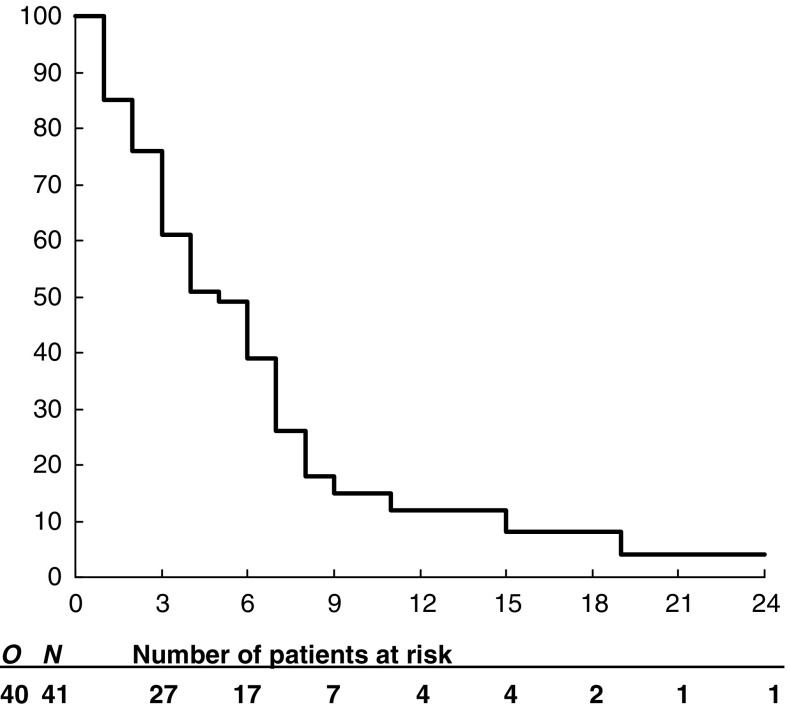
Overall survival. *N*=number of eligible patients. *O*=total number of events at the final analysis. Subsequent numbers are the number of patients at risk.

All patients, apart from one dying from heart failure while PF, had PD. In the five patients without radiological documentation of PD, PFS was calculated as the interval between treatment initiation and death. Median and 6-month PFS was 1.8 months (interquartile range: 1.2–4.5 months) and 14.6% (95% CI 4.6–24.6%; Table 4). A total of 40 patients died. One is alive at 29 months. Median and 1-year OS was 5.2 months (interquartile range: 2.3–7.5 months) and 12.2% (95% CI 2.2–22.2%; Figure 1; Table 4), respectively. Median survival for patients with PR, SD, and PD was 7.4, 6.8, and 2.5 months, respectively. Median survival was 2.9 months for 22 patients without CA19.9 reduction and 7.4 months for 13 patients with CA19.9 reduction >50% (*P*=0.006).

### Quality of life

At baseline, questionnaires were completed by 29 patients (71%). Two of those had PD after the first cycle, while three patients completed only baseline questionnaires. Thus, 24 patients (59%) were assessable for QOL analysis. In this subset of patients, a clinically significant improvement in QOL relative to baseline was observed in health-care satisfaction (50%), body image (42%), fear for future health (40%), pain (39%), sexuality, digestive symptoms (33%), QOL 1 and 2 (30–35%), altered bowel habit and cachexia (30%), cognitive functioning, hepatic symptoms, pancreatic pain (29%), physical functioning, fatigue (26%), nausea, and appetite loss (24%).

### Exploratory analyses

As the aim of salvage treatment in metastatic pancreatic cancer is palliative, exploratory analyses of the impact of TOM-OX on OS in subgroups of patients were performed in an attempt to identify those who could receive the greatest benefit from treatment (significance level after multiple comparison adjustment: 0.0036). Previous chemotherapy including 5-fluorouracil or cisplatin did not reduce the probability to be PF at 6 months or alive at 12 months after TOM-OX (Table 4). When considering patients who received only one prior chemotherapy line, no significant difference in OS was observed among 16 patients previously treated by PEFG when compared to 17 patients treated by gemcitabine alone (1-year OS 25.0 *vs* 0%; *P*=0.018). A summary of univariate and multivariate analyses of the relationship between OS and patient-, treatment-, and tumour-related variables is reported in Table 5. Overall survival was significantly longer in patients with previous PFS ranging between 6.1 and 12 months relative to those with shorter PFS and in patients without pancreatic localisation. A trend towards longer OS was observed in patients submitted to previous surgery. A multivariate analysis by the Cox proportional hazard model confirmed that previous PFS and pancreatic localisation were significantly predictive of survival (Table 5).

## DISCUSSION

The present trial showed that TOM-OX regimen was feasible, had limited toxicity and relevant activity in patients with gemcitabine-resistant metastatic pancreatic adenocarcinoma, and may constitute a treatment opportunity in this setting. It is noteworthy that this regimen was also active in patients with 5-fluorouracil- or cisplatin-resistant disease. Until a decade ago, the use of chemotherapy in pancreatic cancer was believed to have no role in the routine treatment of patients with advanced disease (Lionetto *et al*, 1995). A few options are currently available for first-line treatment (Burris *et al*, 1997; Moore *et al*, 2005; Reni *et al*, 2005). However, gemcitabine-based chemotherapy yields a very limited disease control, and progression usually occurs within a few months after starting first-line treatment. As no further standard therapeutic option exists and scarce information on the impact on outcome of salvage therapy is available, prospective trials attempting to widen the therapeutic armamentarium against this disease are warranted. So far, very few studies have investigated salvage chemotherapy after failure of gemcitabine or gemcitabine-containing chemotherapy (Stehlin *et al*, 1999; Oettle *et al*, 2000; Ulrich-Pur *et al*, 2003; Cantore *et al*, 2004; Milella *et al*, 2004; Reni *et al*, 2004), one of which was retrospective (Kozuch *et al*, 2001). The populations selected were different in terms of proportion of patients with PS>80, which ranged from 0 to 61%, metastatic patients (73–100%), patients with liver metastases (57–85%), patients with >1 prior chemotherapy lines (0–29%), and median PFS after previous treatment (6.0–7.9 months), which was rarely reported in other series, while in our exploratory analyses it resulted as an independent factor predicting the outcome of salvage therapy (Table 6). Furthermore, the sample size of most series is limited to <20 patients per treatment arm (Oettle *et al*, 2000; Ulrich-Pur *et al*, 2003; Milella *et al*, 2004; Reni *et al*, 2004), thus producing data with very large CIs. Given these differences, the lack of information on important prognostic factors, and other potential bias related to phase II trial design, results are difficult to compare across trials, especially in terms of survival. Activity observed in the current trial (PR: 24%) was consistent with the response rate of 16–35% previously reported with other active regimens (Kozuch *et al*, 2001; Ulrich-Pur *et al*, 2003; Milella *et al*, 2004) and compares favourably with the 0–10% objective responses reported elsewhere (Stehlin *et al*, 1999; Oettle *et al*, 2000; Ulrich-Pur *et al*, 2003; Cantore *et al*, 2004; Reni *et al*, 2004). The median PFS of 1.8 months observed with TOM-OX regimen was slightly shorter than the median PFS of 1.7–4.1 months reported in other series (Stehlin *et al*, 1999; Oettle *et al*, 2000; Kozuch *et al*, 2001; Ulrich-Pur *et al*, 2003; Cantore *et al*, 2004; Milella *et al*, 2004; Reni *et al*, 2004). However, it is likely that this depended on the timing of radiographic assessment, which was performed more frequently in the current trial and was therefore more prone to intercept early PD. Consistently, PFS-6 was identical in our series and in the retrospective series which had previously obtained the longest median PFS among published series (Kozuch *et al*, 2001). With regard to grade 3–4 toxicity, neutropenia (12%), nausea/vomiting (7%), and liver enzymes increase (7%) observed in our series were within the range reported with other regimens (5–38, 3–14, and 5–13%, respectively). Fatigue (5%) was reported in a single series (10% (Reni *et al* 2004)). Diarrhoea (2%) was observed less often relative to other series (3–10%). Of note, less toxicity was observed in our series relative to TOM-OX when administered to patients with metastatic colorectal cancer (Cascinu *et al*, 2002; Seitz *et al*, 2002), namely, 17–33% liver toxicity, 10–30% neutropenia, 5–13% nausea-vomiting, 11–16% fatigue, and 7–17% diarrhoea were reported in metastatic colorectal cancer (Cascinu *et al*, 2002; Seitz *et al*, 2002). The differences in toxicity may reflect different selection of patients (e.g. in terms of PS) and may suggest that toxicity profile could be different in different tumour sites. As the aim of salvage therapy in patients with metastatic pancreatic cancer is purely palliative, some concern could be raised that the improvement in clinical outcome is not achieved at the cost of impaired QOL. Clinical benefit response was proposed to address this issue (Burris *et al*, 1997). However, this measure was not validated and has been criticised for using selected variables that do not reflect QOL (Hoffman and Glimelius, 1998). Thus, we preferred a more reliable and validated measure, such as the EORTC QLQ questionnaire. No data to which to compare the present findings are available in the literature. Raltitrexed–oxaliplatin regimen yielded a clinically significant improvement relative to baseline in a large proportion of patients in several QOL domains, including most of the important symptoms that are frequently associated with pancreatic cancer.

Altogether, a median overall survival of 3.5–10 months and median PFS of 2–4 months was achieved with active salvage therapy. It is of note that 12–23% patients with metastatic disease are alive at 1 year from salvage treatment start (current series, Kozuch *et al*, 2001; Ulrich-Pur *et al*, 2003; Cantore *et al*, 2004; Milella *et al*, 2004). These figures are similar to those observed after gemcitabine in the first-line setting. While a bias in favour of salvage therapy due to better selection of patients cannot be ruled out, these data suggest that an appropriately selected subset of patients, for example, on the basis of previous PFS, with gemcitabine-refractory disease may yield a relevant clinical and survival benefit from further treatment. This hypothesis should be tested in a phase III trial against best supportive care.

## Figures and Tables

**Table 1:** Patient characteristics at baseline

**Characteristic**	***n* (%)**
Patients enrolled	41
*Age (years)*	
Median	61
Range	25–80
*Sex*	
Male	23 (56)
Female	18 (44)
*Karnofsky PS*	
70–80	16 (39)
90–100	25 (61)
*Site of metastases*	
Liver	33 (80)
Lymphnodes	8 (20)
Lung	12 (29)
Peritoneum	5 (12)
*Number of metastatic lesions*	
1	1 (2)
2–5	24 (59)
>5	16 (39)
*Prior therapy*	
Prior pancreatic surgery	18 (44)
Prior radiotherapy	14 (34)
*Prior chemotherapy lines*	
*n*=1	35 (85)^*^
*n*>1	6 (15)
*Gemcitabine alone	17 (49)
*Combination	18 (51)

PS=performance status; *n*=number.

* In all, 35 patients received 1 prior chemotheraphy line. Of those, 17 received Gemcitabine alone and 18 received a combination chemotherapy.

**Table 2:** Treatment summary

**Number of cycles**	**Number of patients**
1	6
2	18
3	1
4	1
5	2
6	13

**Table 3:** Treatment-related toxicity per cycle (and worst ever by patient)

**Toxicity**	**Grade 0**	**Grade 1/2**	**Grade 3**	**Grade 4**	**NA**
Granulocytes	66 (63)	22 (22)	3 (5)	3 (7)	6 (2)
Platelets	70 (61)	22 (29)	2 (5)	1 (2)	6 (2)
Haemoglobin	39 (29)	54 (66)	1 (2)	0	6 (2)
Stomatitis	98 (93)	2 (7)	0	0	0
Nausea/vomiting	61 (46)	36 (46)	3 (5)	1 (2)	0
Diarrhoea	90 (76)	9 (22)	1 (2)	0	0
Neurologic	81 (68)	19 (32)	0	0	0
Fatigue	72 (49)	27 (46)	2 (5)	0	0
Liver (GOT/GPT)	62 (49)	30 (41)	2 (5)	1 (2)	6 (2)
Liver (GGT/Alk.P)	88 (83)	5 (12)	1 (2)	0	6 (2)
Fever	92 (80)	8 (20)	0	0	0
Kidney	98 (93)	2 (7)	0	0	0

Numbers are expressed as percentages. NA=not available.

**Table 4:** Activity and efficacy analyses summary

	**Best response**		**Outcome measures**	
**Previous treatment**	**PR**	**SD**	**PFS-6**	**OS-12**
All patients (*n*=41)	10 (24.4%)	11 (26.8%)	6 (14.6%)	5 (12.2%)
PEFG (*n*=16)	3 (18.7%)	6 (37.5%)	3 (18.8%)	4 (25.0%)
G (*n*=17)	5 (29.4%)	4 (23.5%)	2 (11.8%)	0 (0.0%)
G → PEFG (=5)	0 (0.0%)	1 (20.0%)	0 (0.0%)	0 (0.0%)
*F including*				
y (*n*=23)	5 (21.7%)	7 (30.4%)	4 (17.4%)	5 (21.7%)
n (*n*=18)	5 (27.8%)	4 (22.2%)	2 (11.8%)	0 (0.0%)
*P including*				
y (*n*=22)	3 (13.6%)	7 (31.8%)	3 (13.6%)	4 (18.2%)
n (*n*=19)	7 (36.8%)	4 (21.1%)	3 (15.8%)	1 (5.3%)
*n of lines*				
1 (*n*=35)	9 (25.7%)	10 (28.6%)	6 (17.1%)	5 (14.3%)
>1 (*n*=6)	1 (16.6%)	1 (16.6%)	0 (0.0%)	0 (0.0%)

*n*=number; PR=partial response; SD=stable disease; PFS-6=progression-free at 6 months; OS-12=alive at 12 months; P=cisplatin; E=epirubicin; F=5-fluorouracil; G=gemcitabine; y=yes; n=no; → =followed by.

**Table 5:** Exploratory analyses summary

				**Univariate**		**Multivariate**	
**Variable**	**Subgroups**	**No. of patients**	**1 year OS (%)**	***P***	**HR**	**95% CI**	***P***
PFS	<6 months	22	0.0				
	⩾6 months	19	21.1	0.0035	4.27	1.56–11.67	0.007
							
Surgery	Yes	18	22.2				
	No	23	0.0	0.0049	2.26	0.48–10.79	0.31
							
CHT lines	1	6	11.4				
	>1	35	0.0	0.1278	1.49	0.38–5.83	0.58
							
Age	⩽60	19	15.8				
	>60	22	4.5	0.3362	1.27	0.50–3.21	0.62
							
Gender	Male	23	17.4				
	Female	18	0.0	0.0296	0.52	0.23–1.19	0.13
							
PS	90–100	25	12.0				
	70–80	16	6.2	0.2882	1.29	0.49–3.38	0.61
							
Radiotherapy	Yes	14	11.1				
	No	27	7.1	0.3601	0.78	0.34–1.80	0.56
							
No. of lesions	2–5	24	4.2				
	>5	16	18.8	0.4529	0.62	0.18–2.12	0.45
							
Site: liver	Yes	33	9.1				
	No	8	12.5	0.4891	0.96	0.32–2.88	0.94
							
Site: lung	Yes	12	16.7				
	No	29	6.9	0.4457	1.06	0.31–3.58	0.93
							
Site: pancreas	Yes	26	0.0				
	No	15	26.7	0.0011	8.46	1.34–53.4	0.03
							
Site: peritoneum	Yes	5	0.0				
	No	36	11.1	0.3860	0.37	0.08–1.76	0.22
							
No. of sites	1	7	42.9				
	>1	34	2.9	0.0080	0.70	0.14–3.42	0.66

No.=number; CHT=chemotherapy; PFS=progression-free survival; OS=overall survival; PS=performance status; HR=hazard ratio; CI=confidence interval.

**Table 6:** Results of salvage therapy for pancreatic adenocarcinoma

**Ref**	**No. of pts**	**Treatment**	**m. age**	**PS 0**	**M (%)**	**liver M (%)**	**>1CHT (%)**	**PPFS**	**ORR (%)**	**mPFS**	**PFS-6 (%)**	**1 year OS (%)**
7	30	I+E	60	30	100	60	23	Nr	10	4.1	Nr	23
15^a^	34	G-FLIP	64	Nr	100	85	29	Nr	24	3.9	20	20
17	17	F+celecoxib	60	35	82	Nr	0	Nr	35	1.9	6	20
21	18	T	59	Nr	100	Nr	22	7.9	5.5	3.3	Nr	Nr
26	15	MDI	61	26	100	60	20	Nr	0	1.7	0	0
29	33	Ru	62	0	73	57	Nr	Nr	9	Nr	Nr	6
30	19	R	60	21	100	74	0	Nr	0	2.5	Nr	0
30	19	R+I	63	21	100	63	0	Nr	16	4.0	Nr	22
cs	41	R+E	61	61	100	80	15	6.0	24	1.8	15	12

Ref=reference; No. of pts=number of patients; m. age=median age; PS 0=performance status=0 (ECOG) or 90–100 (Karnofsky); M=metastatic; CHT=% of patients with >1 previous chemotherapy lines; PPFS=previous progression-free survival; ORR=objective response rate; m PFS=median progression-free survival; PFS-6=progression-free survival at 6 months; 1 year OS=overall survival at 1 year; cs=current series; I=irinotecan; E=eloxatin; G=gemcitabine; F=5-fluorouracil; L=leucovorin; P=cisplatin; T=paclitaxel; M=mitomycin; D=docetaxel; Ru=rubitecan; R=raltitrexed; Nr=not reported.

a Retrospective.
